# Turning Work Challenges into Creativity: The Joint Role of Challenge Stress and Problem-Solving Pondering

**DOI:** 10.3390/bs16091625

**Published:** 2026-09-11

**Authors:** Jing Zhang, Yidan Hong, Yiming Lu, Andrew P. Smith, Feng Liu

**Affiliations:** 1 Social Governance Innovation Research Center, Henan Agricultural University, Zhengzhou 450046, China; zhangjingvip98@163.com (J.Z.);; 2 School of Marxism, Hainan College of Foreign Studies, Wenchang 571321, China; nizai88@126.com; 3 Centre for Occupational and Health Psychology, School of Psychology, Cardiff University, Cardiff CF10 3AS, UK

**Keywords:** challenge stress, cognitive flexibility, problem-solving pondering, response surface, work creativity

## Abstract

Previous studies have yielded inconsistent conclusions regarding the relationship between challenge stress and work creativity, suggesting that the effects may depend on contextual and individual conditions. Drawing on the strategy–situation fit hypothesis, this study examines the joint effects of challenge stress and problem-solving pondering on work creativity and further investigates the mediating role of cognitive flexibility. Using a two-wave time-lagged design with a two-month interval, this study ultimately obtained 568 valid responses. The results showed the following: (1) Work creativity was higher when challenge stress and problem-solving pondering were jointly high than when both were low, as indicated by the significant positive slope along the CS = PSP reference line. The curvature along the reference line was nonsignificant, indicating that creativity increased approximately linearly as the two variables increased simultaneously. Along the CS = −PSP reference line, a significant asymmetrical, U-shaped misfit effect emerged. Specifically, creativity was severely hindered when challenge stress exceeded problem-solving pondering, whereas the decline was less pronounced when problem-solving pondering exceeded challenge stress. These findings indicate that work creativity varies across different joint configurations of challenge stress and problem-solving pondering. (2) Cognitive flexibility mediated the relationship between the joint effects of challenge stress and problem-solving pondering and creativity (indirect effect *β* = 0.17, 95% *CI* [0.0923, 0.2931]). The findings suggest that higher joint levels of challenge stress and problem-solving pondering are associated with greater cognitive flexibility, which may in turn contribute to greater creativity. Organizations seeking to enhance employees’ creative performance should not focus solely on increasing external challenges but should also cultivate employees’ problem-solving pondering and cognitive flexibility.

## 1. Introduction

Work creativity has long been a central concern in modern society. In today’s era of rapid technological advancement, fostering workplace innovation remains a key driver of organizational growth. Among the various factors influencing work creativity, work stress has received considerable scholarly attention. In particular, the relationship between challenge stress and work creativity is complex, with existing studies reporting negative, positive, nonlinear, or even nonsignificant associations. To account for these inconsistent findings, researchers have examined this relationship from multiple perspectives—such as the types of challenges, levels of stress intensity, and boundary conditions—to develop a more comprehensive understanding of how challenge stress affects creativity at work. Among these efforts, two meta-analyses have examined the relationship in depth. A 2010 meta-analysis emphasized the social evaluative threats posed by various stressors and highlighted the crucial role of individual factors in shaping how people perceive and respond to stress. It suggested incorporating individual characteristics into analytical models investigating the link between stressors and creativity (Byron et al., 2010). The more recent 2025 meta-analysis by Huang and Yu found that, overall, stress exerts a significant negative impact on creativity. However, when stress was further subdivided, the findings became more nuanced: different levels of stress influenced work creativity in distinct ways. Specifically, mild social evaluative threats (SETs)—those involving a single social-evaluative element—slightly but not significantly enhanced creativity, whereas severe SETs, involving three social-evaluative elements, significantly inhibited creativity, suggesting a curvilinear relationship between SETs and creativity. Furthermore, challenge stress had no significant effect on creativity (Huang & Yu, 2025).

Collectively, these findings indicate that it is difficult to draw a single, consistent conclusion regarding the relationship between challenge stress and work creativity. As Byron et al. (2010) observed, it is overly simplistic to claim that stress exerts merely positive or negative effects on creativity. Instead, future research should focus on how to enhance employees’ creative performance under conditions of challenge stress—specifically, by identifying the boundary conditions that enable stress to foster creativity rather than hinder it.

Previous research examining the mechanisms that moderate the effects of challenge stress has largely centered on organizational and leadership factors, such as organizational support (Wallace et al., 2009), supervisor support (Buch et al., 2015), superior–subordinate relationships (X. Zhang et al., 2017), and leadership styles (Y. Zhang et al., 2014). Compared with these external factors, employees’ internal characteristics—including their skills, abilities, and behavioral tendencies—are generally more stable and manageable. Consequently, recent studies have emphasized the importance of incorporating individual differences into models of the stress–creativity relationship, as such differences help explain the varied ways individuals perceive and respond to stress. Therefore, rather than focusing exclusively on external environmental factors, greater attention should be directed toward understanding the internal psychological conditions that shape employees’ creative responses to challenge stress.

According to the strategy–situation fit hypothesis, selecting appropriate coping strategies based on the controllability of stressors can significantly influence coping outcomes (Cheng et al., 2014). Under conditions of challenge stress, problem-focused coping is more effective than emotion-focused coping (Person & Frazier, 2024). Building on this perspective, this study examines how individual strategies shape the conditions under which employees display higher levels of creativity in response to challenge stress.

## 2. The Joint Effects of Challenge Stress–Problem-Solving Pondering on Work Creativity

Existing research shows that when employees encounter work-related stress, they may engage in two distinct forms of work-related rumination: one centered on emotional experiences and the other on problem-solving. The latter, characterized by persistent cognitive engagement with work difficulties, is known as problem-solving pondering (Cropley & Zijlstra, 2011). This form of rumination reflects deliberate, sustained mental effort to analyze specific work issues or evaluate past performance to identify potential improvements. It is inherently goal-oriented, encompassing behaviors such as reexamining problems from alternative perspectives, identifying and eliminating barriers, and developing innovative solutions.

Previous studies have consistently shown that problem-solving pondering positively affects both individual well-being and work outcomes. For instance, engaging in problem-solving pondering during non-work hours has been found to significantly reduce both chronic and acute fatigue on subsequent workdays (Querstret & Cropley, 2012). Similarly, positive reflections on work during leisure time predict higher levels of proactive behavior and organizational citizenship behavior, thereby enhancing employees’ overall well-being (Binnewies et al., 2009; Fritz & Sonnentag, 2009). More importantly, problem-solving pondering has been shown to exert a significant positive impact on work creativity. Through continuous redefinition and exploration of work-related issues, individuals gain new insights and learn from prior problem-solving experiences. Weinberger et al. (2018) found that entrepreneurs’ problem-solving pondering during the evening significantly and positively predicted their creative performance the following day. Likewise, cross-sectional research across various industries has confirmed that employees’ problem-solving pondering positively predicts work creativity (J. Zhang et al., 2021), and this beneficial effect even persists over the course of one year (Vahle-Hinz et al., 2017).

Therefore, when encountering various workplace challenges, individuals who engage in problem-solving-oriented pondering are more likely to enhance their work creativity under stress. Grounded in the strategy–situation fit hypothesis, for controllable stress (i.e., challenge stress), adopting problem-focused coping strategies is associated with better mental health (Cheng et al., 2014). In contrast, in controllable stress situations, emotion-focused coping strategies may undermine well-being (Haines et al., 2016). Intervention studies have further shown that enhancing individuals’ use of coping strategies aligned with specific stress contexts—namely, adopting problem-focused coping under challenge stress conditions and emotion-focused coping under hindrance stress conditions—can effectively reduce depressive symptoms (Cheng et al., 2007). Consequently, when work challenges interact synergistically with employees’ problem-solving pondering—that is, when individuals adopt a problem-solving cognitive style to address stressors—they are more likely to exhibit positive adaptive responses and greater creative performance.

When challenge stress is low, individuals often lack sufficient work motivation, which dampens their drive to transcend established routines and explore novel solutions. Although challenging demands inherently increase workplace pressure, moderate levels of such stressors can activate approach-oriented motivation (Cavanaugh et al., 2000), prompting employees to invest greater cognitive resources into resolving task difficulties. Conversely, under low-challenge conditions, work demands remain within the comfort zone of individuals’ capabilities and resources; consequently, employees are predisposed to rely on existing knowledge and experience rather than pursuing exploratory experimentation.

Simultaneously, a deficiency in problem-solving pondering minimizes the likelihood of sustained reflection on potential solution pathways and the efficacy of alternatives to workplace issues. This deficit restricts opportunities for the creative recombination of existing information. Under the joint configuration of low challenge stress and low problem-solving pondering, individuals lack both the situational impetus for innovation and the cognitive processing facilitated by sustained reflection. They are therefore more likely to rely on familiar thought patterns and routine behavioral approaches when dealing with work tasks.

Based on the above discussion, this study proposes:

**Hypothesis 1 (H1).**  *Challenge stress and problem-solving pondering jointly influence work creativity such that creativity is expected to be higher when both challenge stress and problem-solving pondering are simultaneously high than when both are simultaneously low.*

## 3. The Mediating Role of Cognitive Flexibility

Cognitive flexibility refers to an individual’s ability to adapt cognitive patterns in response to continuously changing environmental stimuli (Dennis & Vander Wal, 2010). Individuals with high cognitive flexibility tend to actively seek information that challenges their existing viewpoints, maintain an open attitude toward new information and ideas (Martin et al., 2011), and adopt strategies that align with a changing environment (Kiss et al., 2020). Such individuals can assimilate and accommodate new knowledge, thereby constructing novel cognitive frameworks. In stressful work situations, their openness and diverse problem-solving approaches enable them to integrate new environmental demands with existing practices, identify innovative improvement opportunities, and actively adjust strategies, ultimately leading to enhanced work performance and creativity (Stad et al., 2019; Lange et al., 2015).

Researchers have noted that individuals’ effective attention is significantly enhanced under challenge states. This process directs attention toward task-related cues in an approach-oriented manner. Such an approach motivational state can facilitate the goal-advancing and cognitive processes involved in coping flexibility, thereby improving one’s capacity to evaluate and select effective strategies based on situational characteristics—all of which manifest cognitive flexibility (Chen & Qu, 2023). Further experimental studies have revealed that cognitive flexibility is not necessarily enhanced under challenge stress conditions; rather, a more critical determinant is an individual’s mindset toward challenge stress (Crum et al., 2017). Specifically, cognitive flexibility is enhanced only when challenge stress is met with a growth-oriented mindset; conversely, a negative mindset can cause challenge stress to exert a detrimental effect on cognitive flexibility (Crum et al., 2017; Chen & Qu, 2023). When individuals engage in problem-solving pondering to cope with challenge stress, they may be more likely to perceive the current challenge as manageable and potentially controllable. This is because challenge stress typically involves demanding yet potentially attainable goals and opportunities for growth and achievement. By continuously exploring possible solutions and considering alternative courses of action, individuals may develop a greater sense of control over the stressor and believe that they can effectively manage the demands they encounter. Therefore, the synergistic relationship between challenge stress and problem-solving pondering is expected to facilitate cognitive flexibility.

Based on the above, this study proposes:

**Hypothesis 2 (H2).**  *Cognitive flexibility mediates the relationship between the joint effects of challenge stress and problem-solving pondering and work creativity.*

## 4. Research Process and Methods

### 4.1. Sample and Procedure

To reduce common method bias, data were collected at two time points (Zhou & Long, 2004). Control variables, challenge stress, and problem-solving pondering were collected at Time 1 (T1), while cognitive flexibility and work creativity were measured two months later (T2). A snowball sampling approach was employed: research assistants initially distributed online questionnaires to full-time employees across various industries and encouraged participants to forward the surveys to their colleagues. Participants were coded using a combination of surname and date of birth to ensure anonymity and facilitate matching across time points. Those who completed only the first measurement received one-third of the compensation, while participants completing both measurements received the full payment.

The data from the two measurement waves were matched, yielding a final effective sample of 568 participants from 34 provinces in China. The mean age of all participants is *M ± SD* (36.82 ± 10.50). The mean weekly working hours are *M ± SD* (45.04 ± 8.65). The average tenure at the current organization is *M ± SD* (11.16 ± 10.64). The sample comprised 245 males (43.10%) and 323 females (56.90%), with 231 (40.70%) having a junior college degree or below, 303 with a bachelor’s degree (53.30%), and 34 with a master’s degree or above (6.00%). Participants worked in a range of settings: 86 (15.14%) in state-owned enterprises, 208 (36.62%) in non-state-owned enterprises, 255 (44.89%) in governmental agencies or public institutions, and 19 (3.35%) in other occupational settings. Among the respondents, 231 (40.70%) have children under 18 who require care, while 337 (59.30%) do not. A total of 309 (54.40%) has elderly family members assisting with household chores, whereas 259 (45.60%) do not.

### 4.2. Measures

The measures used in this study have all been employed in previous research, demonstrating adequate reliability and construct validity. Following Brislin’s (1970) back-translation procedure, the original survey items were translated into Chinese and then back-translated into English. The translators and study investigators resolved any discrepancies between the original and back-translated versions, ensuring conceptual equivalence across languages. The standardized factor loadings for all scales ranged from 0.56 to 0.90.

Challenge stress. This was measured using the challenge stress subscale of the Challenge-Hindrance Stress Scale developed by Cavanaugh et al. (2000). Six items were used to measure challenge stress (e.g., “The number of projects or tasks you are responsible for”). Participants rated the degree to which the situation described in each item caused stress using a five-point Likert scale from 1 (no stress) to 5 (a great deal of stress). In the current study, Cronbach’s alpha of the challenge stress subscale was 0.91, and the AVE was 0.64.

Cognitive flexibility. This was measured with the Cognitive Flexibility Inventory developed by Dennis and Vander Wal (2010) using a seven-point Likert scale from 1 (strongly disagree) to 7 (strongly agree). This study retained the items from the original study whose factor loadings were above 0.50 across both measurement waves. In the end, eight items were retained to measure cognitive flexibility. An example of the items is “I can think of more than one way to solve the problems I face”. In the current study, Cronbach’s alpha was 0.96 for cognitive flexibility, and the AVE was 0.74.

Problem-solving pondering. This was measured with a five-item questionnaire developed by Cropley et al. (2012) on a five-point Likert scale from 1 (strongly disagree) to 5 (strongly agree). The example item is “I find solutions to work-related problems in my free time.” In the current study, Cronbach’s alpha for the problem-solving pondering scale was 0.88, and the AVE was 0.59.

Work creativity. Employee creativity was assessed using four items from Tierney et al. (1999) on a 6-point scale from 1 (strongly disagree) to 6 (strongly agree). An example item is “I try new ideas or methods first.” In the current study, the scale’s Cronbach’s alpha was 0.90, and the AVE was 0.70.

Control variables were gender, age, average tenure at the current organization, education level (1 = junior college degree or below; 2 = bachelor’s degree; 3 = master’s degree or above), job type (1 = state-owned enterprises; 2 = non-state-owned enterprises; 3 = governmental agencies or public institutions; 4 = other occupational settings), whether they had children under 18 who require care, whether elderly family members assisted with household chores, and average working hours per week. 

## 5. Results

### 5.1. Descriptive Statistics and Correlation Analysis of Research Variables

An analysis was conducted on the correlations between the research variables: challenge stress (T1), problem-solving pondering (T1), cognitive flexibility (T2), and work creativity (T2). The results showed (See Table 1) that challenge stress was significantly correlated with both problem-solving pondering and cognitive flexibility (*p* < 0.01). In addition, both problem-solving pondering and cognitive flexibility were significantly correlated with work creativity (*p* < 0.01).

### 5.2. Assessment of Common Method Variance and Confirmatory Factor Analysis

The data in the present study were collected via self-administered questionnaires. Therefore, common method variance could inflate the strength of observed relationships (Podsakoff et al., 2003). Two methods were used to reduce the impact of common method variance. First, Harman’s single-factor test was used to determine whether each measure explained unique variance in the data. Exploratory Factor Analysis showed there were four factors (λ > 1) when there was no rotation. The first factor explained 33.23% of the variance, which did not account for most of the covariance among the measures. Second, confirmatory factor analysis was performed on the data. The results showed that the fit of the single-factor model (χ^2^/*df* = 15.82, CFI = 0.64, TLI = 0.59, GFI = 0.61, RMSEA = 0.16) was poor. In sum, the results from this study cannot be solely attributed to common method variance.

Additionally, as shown in Table 2, the fit index of the proposed four-factor model significantly outperformed those of the other three alternative models, indicating that the hypothesized model is a better fit for the data in this study and that the four factors are mutually independent and have good discriminant validity.

### 5.3. Hypothesis Testing

Based on previous research, the relationship between challenge stress and work creativity is complex and cannot be adequately captured using a simple linear model. Quadratic polynomial regression combined with response surface analysis (Freeny, 1988)—commonly referred to as the quadratic response surface regression method—is particularly suited for examining such relationships from the perspective of a strategy–situation fit hypothesis. This approach allows rigorous analysis of both the individual effects of two predictors and the interaction between them (Edwards, 2008). Furthermore, response surface analysis enables the creation of a three-dimensional visual representation based on the regression coefficients derived from the quadratic polynomial regression, offering a more intuitive and interpretable depiction of the data.

Data analysis was conducted using SPSS 21.0. First, descriptive statistics were computed in SPSS 21.0, and the polynomial regression effect of the joint interaction between challenge stress and problem-solving pondering on creativity was analyzed. Then, the SPSS macro program compiled by Hayes (2013) was run to test the mediating effect of cognitive flexibility.

#### 5.3.1. Joint Effects Analysis

The analysis used a combination of polynomial regression and response surface methodology. Specifically, work creativity (C) was regressed on challenge stress (CS), problem-solving pondering (PSP), the interaction between challenge stress and problem-solving pondering (CS × PSP), the square of challenge stress (CS^2^), and the square of problem-solving pondering (PSP^2^). The equation is expressed as follows:

C = b_0_ + b_1_ CS + b_2_ PSP + b_3_ CS^2^ + b_4_ CS × PSP + b_5_ PSP^2^ + e.

**Figure 1:**
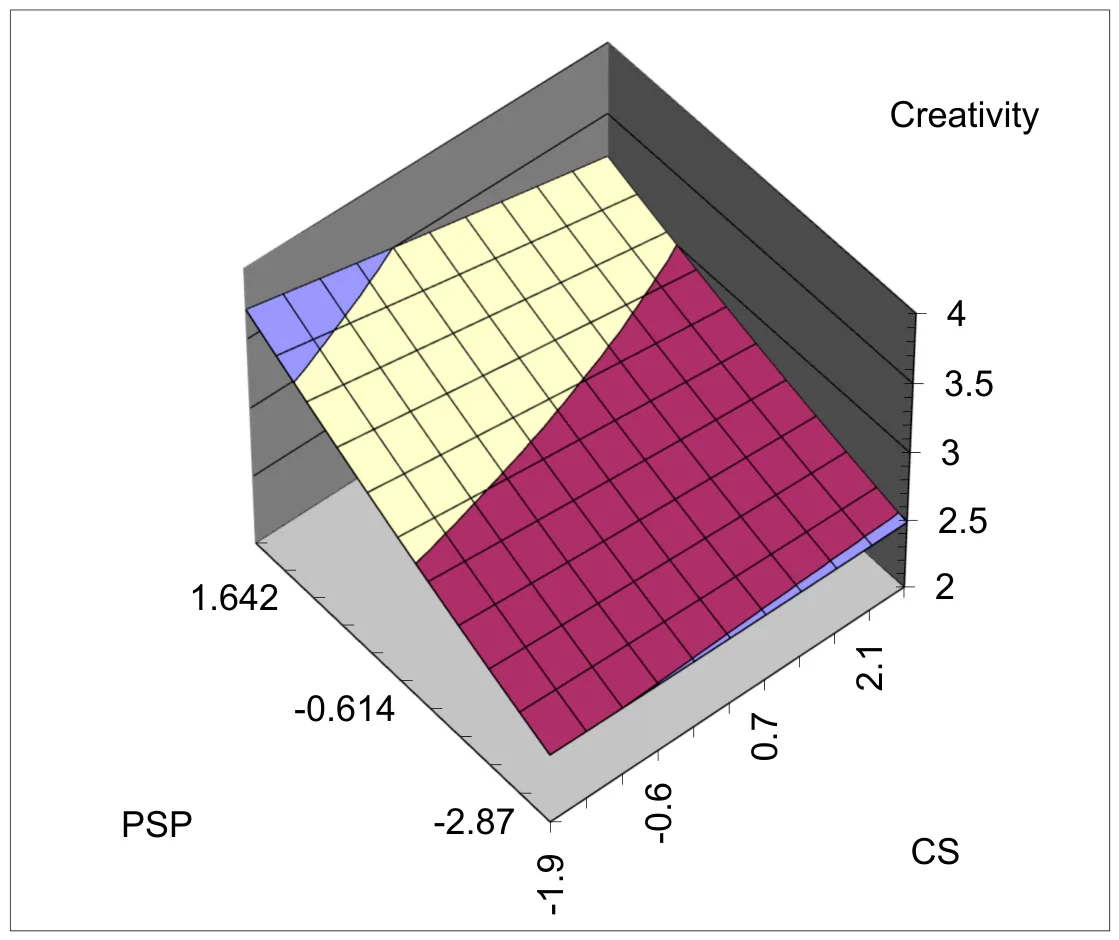
Response surface of the joint effects of challenge stress (CS) and problem-solving pondering (PSP) on creativity.

To avoid multicollinearity, all variables were centered. A hierarchical regression approach was employed, with control variables entered in the first step (M1); challenge stress and problem-solving pondering entered in the second step to test their main effects (M2); and the squared terms of challenge stress and problem-solving pondering, along with their interaction term, entered in the third step (M3). The specific details are presented in Table 3 and Figure 1. The reference lines conventionally denoted as CS = PSP and CS = −PSP were used to facilitate the interpretation of patterns in which the two predictors change together or differ relative to one another.

The polynomial regression results showed that the ΔR^2^ of Model 3 (M3) was significant at the 0.01 level, indicating that the data were suitable for polynomial regression analysis. The results indicated that along the CS = PSP reference line, the slope was significantly positive (a1 = 0.16, *p* < 0.01) while the curvature was nonsignificant (a2 = −0.02, *p* = 0.069), suggesting that when the two variables are congruent, creativity increases linearly as they jointly increase, meaning that the “high–high joint” condition yields a significantly better effect than the “low–low joint” condition. Conversely, along the CS = −PSP reference line, the slope was significantly negative (a3 = −0.27, *p* < 0.001) and the curvature was significantly positive (a4 = 0.03, *p* < 0.01), demonstrating that incongruence exerts a significant U-shaped curvilinear effect on creativity. On the one hand, when challenge stress relatively exceeded problem-solving pondering (CS > PSP), creativity was comparatively low; on the other hand, creativity declined less sharply when problem-solving pondering exceeded challenge stress (PSP > CS), suggesting that higher levels of problem-solving pondering may partially offset the adverse consequences associated with insufficient challenge stress.

Overall, these results confirm that challenge stress and problem-solving pondering jointly shape work creativity, such that work creativity is higher when both challenge stress and problem-solving pondering are high than when both are low. The effects of challenge stress and problem-solving pondering on creativity are asymmetric and nonlinear, depending on both their joint levels and the direction of their relative difference. The response surface results were consistent with Hypothesis 1.

#### 5.3.2. Mediating Effect Analysis

Following the polynomial block variable approach proposed by Edwards and Parry (1993), the block variable was constructed to integrate the joint effects captured by the five polynomial terms while preserving the explanatory information contained in the original polynomial regression model. First, challenge stress (CS) and problem-solving pondering (PSP) were mean-centered, and five polynomial terms were subsequently constructed (CS, PSP, CS^2^, CS × PSP, PSP^2^). Then, a polynomial regression analysis was conducted with cognitive flexibility as the dependent variable. The results showed that the ΔR^2^ of Model 3 (M3) was significant at the 0.05 level, supporting the first stage of the proposed mediation model. Subsequently, a block variable was constructed by weighting the five polynomial terms using unstandardized regression coefficients obtained from the polynomial regression model [unstandardized coefficients: CS (*B* = 0.07, SE = 0.07), PSP (*B* = 0.42, SE = 0.10), CS^2^ (*B* = 0.02, SE = 0.01), CS × PSP (*B* = −0.04, SE = 0.02), PSP^2^ (*B* = 0.02, SE = 0.02)]. Finally, a mediation analysis using the PROCESS macro with the bootstrap method was conducted (1000 iterations).

The results (Table 4) showed that a block of challenge stress–problem-solving pondering significantly and positively predicted cognitive flexibility (*B* = 1.00, *p* < 0.001) and significantly predicted creativity (*B* = 0.19, *p* < 0.05), while cognitive flexibility significantly and positively predicted creativity (*B* = 0.18, *p* < 0.001). This indicates that cognitive flexibility plays a significant mediating role between the block of challenge stress, problem-solving pondering, and creativity, with an indirect effect size of (*β* = 0.17, 95% *CI* [0.0923, 0.2931]). The total effect of block on creativity was (*β* = 0.36, 95% *CI* [0.2020, 0.5258]), and the direct effect of block on creativity was (*β* = 0.19, 95% *CI* [0.0329, 0.3361]). Therefore, hypothesis 2 was supported.

## 6. Discussion

Although work innovation has long been a focal topic for scholars, many have noted that the existing body of research remains insufficient (Luis & Ruth, 2020). Some researchers argue that stress can hinder creativity because it places high demands on cognitive resources, reducing individuals’ willingness to engage in tasks that require significant cognitive investment (Eysenck, 1995). Consequently, individuals may adopt simplified cognitive strategies, resulting in fewer innovative ideas (Baron, 1986). However, other scholars argue that stress can enhance creativity, proposing that under stressful conditions, individuals may be motivated to solve problems and seek innovative solutions actively (Anderson et al., 2004). Empirical studies support this perspective, showing that stress can indeed have positive effects on creative performance. For instance, Ren and Zhang (2015) investigated the impact of challenge stress on creativity. They found that stressors such as urgency, heavy workload, and job responsibilities were positively associated with both idea generation and implementation.

A substantial body of research has demonstrated that the relationship between challenge stress and work creativity cannot be easily categorized as simply positive or negative. Consequently, the critical question is not whether challenge stress promotes or inhibits creativity, but under what conditions it can exert a positive influence. Existing studies addressing this question generally fall into three types. The first type examines the nonlinear relationship between challenge stress and work creativity. The second type focuses on specific subtypes of work challenges, investigating how different stressors may affect creativity differently. The third type considers third-party factors, such as contextual or moderating variables, to better understand the boundary conditions that shape the challenge stress–creativity relationship.

Some researchers have analyzed the effects of stress intensity on creativity, finding that occupational stress is detrimental to idea generation to some extent, but that beyond a certain point, it can enhance innovative behavior (Luis & Ruth, 2020). However, the specific inflection points at which challenge stress begins to enhance creativity have yet to be systematically investigated. Studies examining different types of stress have contributed to understanding how specific stressors affect work creativity. However, the findings remain inconsistent. For example, some research indicates that time pressure significantly reduces creativity (Huang & Yu, 2025), whereas other studies report no significant relationship between perceived time pressure and creativity (Amabile & Gryskiewicz, 1989; Rasulzada & Dackert, 2009).

A more effective approach is to examine the relationship between challenge stress and creativity by incorporating third-party conditions, as this allows researchers to identify the circumstances under which challenge stress produces positive effects. However, previous studies have primarily examined the conditions under which challenge stress exerts its positive effects from a moderation perspective. These moderating factors have mainly focused on organizational-level variables, including support from the organization (Wallace et al., 2009) or supervisors (Buch et al., 2015), developmental feedback from supervisors (Liu et al., 2023), and leadership styles (Y. Zhang et al., 2014). For employees, these situational factors are less controllable. In contrast, the problem-solving pondering examined in the present study is essentially a coping style for dealing with stress, which is more malleable and can be enhanced through various training programs (Ayres & Malouff, 2007). Therefore, it may have greater practical implications. Furthermore, the quadratic polynomial response surface analysis employed in this study not only enables the investigation of the direct effects of work stress on creativity but also provides a more nuanced understanding of how creativity varies across different joint configurations of challenge stress and problem-solving pondering (Edwards, 2008). This approach enables the identification of potential nonlinear relationships that traditional moderation analyses cannot capture. In other words, the perspective and methodology adopted here provide a more comprehensive and systematic framework for understanding the complex relationship between work challenges and creativity.

The results support Hypothesis 1 regarding the joint effect of challenge stress and problem-solving pondering on employee creativity. As indicated by the significant positive slope along the CS = PSP reference line, creativity was lowest when both challenge stress and problem-solving pondering were low, and it increased linearly as the two variables increased simultaneously. Meanwhile, the non-significant curvature along this line indicated that this positive relationship was approximately linear. This finding suggests that employees may be more likely to demonstrate creativity when under the “high–high” joint condition.

Challenge stress provides employees with demanding and potentially stimulating work situations that require new solutions, whereas problem-solving pondering enables employees to analyze work problems and continuously explore alternative solutions. Together, these conditions may provide the impetus and cognitive processing needed for creative performance.

Along the CS = −PSP reference line, both the slope and the curvature were statistically significant. The significant negative slope indicates a pronounced asymmetry: creativity was substantially lower when challenge stress exceeded problem-solving pondering (CS > PSP) relative to matched levels, because employees face high work demands without sufficient problem-focused coping resources. In contrast, when problem-solving pondering exceeded challenge stress (PSP > CS), the decline in creativity was less sharp, suggesting that active problem-solving engagement may partially offset the adverse consequences associated with insufficient challenge stress. The significant positive curvature further reveals that the surface exhibits an asymmetrical U-shaped pattern along the incongruence line. Overall, these results demonstrate that the degree and direction of mismatch between challenge stress and problem-solving pondering systematically shape work creativity, with the most detrimental outcomes occurring when work challenges substantially exceed individual problem-solving strategies. Based on these findings, an important factor associated with whether work challenges are positively associated with work creativity is an individual’s engagement in problem-solving pondering. The strategy–situation fit hypothesis suggests that problem-solving coping is particularly appropriate when individuals encounter relatively controllable, goal-relevant stressors such as challenge stress (Cheng et al., 2014). While previous studies have confirmed that the alignment between the two promotes well-being (Haines et al., 2016) and health (Cheng et al., 2007; Cheng et al., 2014), this study further demonstrates that the joint effect between challenge stress and problem-solving pondering is pivotal for employee creativity. This finding also validates prior research suggesting that the effects of stressors on creative performance depend on how individuals experience and cognitively respond to stress (Byron et al., 2010). When employees cope with work challenges through problem-solving pondering, they may be more likely to frame stress as controllable and resolvable, enabling them to generate creativity driven by challenge stress (Sacramento et al., 2013).

This study also found that cognitive flexibility mediated the relationship between the joint effects of challenge stress and problem-solving pondering on work creativity. Cognitive flexibility refers to an individual’s ability to shift flexibly between tasks. It facilitates actively seeking information to cope with changing environmental stimuli and can be influenced through various activities (Rathore & Lom, 2017; Bassis et al., 2023). According to the strategy–situation fit hypothesis (Cheng et al., 2014), problem-solving pondering may facilitate a more constructive appraisal of challenge stress, potentially increasing employees’ perceptions of its controllability. Under this positive stress mindset, stress is positively associated with cognitive flexibility (Crum et al., 2017; Chen & Qu, 2023); meanwhile, individuals who cope with work challenges through problem-solving pondering are effectively engaging in a form of cognitive training that activates the prefrontal neural mechanisms of the brain (Benikos et al., 2013). This repeated activation promotes cognitive flexibility, which, in turn, enhances creativity at work.

In short, our results respond to the ongoing calls within challenge stress research to uncover the underlying conditions that unlock its positive potential. This study successfully addresses this gap by specifying problem-solving pondering as an adaptive, problem-focused coping mechanism that effectively actualizes the beneficial outcomes of challenge stress.

## 7. Contributions

Theoretical contributions: Previous research on the challenge stress–creativity relationship has largely operated under two implicit assumptions: (1) that the effects of challenge stress can be adequately understood through moderation models focused primarily on external resources (e.g., organizational or supervisory support), and (2) that challenge stress, when present, is generally beneficial or at least neutral for creativity once certain boundary conditions are met.

Our findings challenge both assumptions. By integrating the strategy–situation fit hypothesis with polynomial response surface analysis, we demonstrate that the joint configuration of challenge stress and employees’ problem-solving pondering determines creative outcomes. In doing so, the study moves beyond treating individual differences merely as moderators and instead positions problem-solving pondering as a core, malleable coping strategy that co-defines the meaning and consequences of challenge stress. This advances the challenge–hindrance literature by incorporating a fit-based, configurational perspective and highlights the need for future research to examine joint-configuration effects rather than main or simple interaction effects alone.

Practice implications: The results of this study suggest that organizations should not simply increase challenge stress, but should maintain appropriate and manageable levels of job demand while protecting employees’ cognitive resources and supporting the use of constructive problem-solving strategies. Secondly, the results showed that cognitive flexibility plays a critical mediating role in the model. Therefore, human resource departments should regularly conduct training programs, such as experiential perspective-taking training, to help employees break habitual thinking patterns across contexts, enhance their cognitive flexibility, and thereby promote workplace innovation. Meanwhile, employees themselves should also consciously engage in self-training to strengthen their professional value.

## 8. Limitations and Future Directions

Participants: Employees in this study were from a range of industries. Future research could more narrowly target occupations that place higher demands on creativity, such as the IT and design sectors, or specific professional groups, including corporate managers and R&D personnel.

Methodologically: First, although collecting data at two time points can mitigate common method bias to some extent, the mediating and outcome variables were not collected separately. Future studies should collect data at three different time points, which could strengthen causal inference. Additionally, because employees self-reported on all variables, there is potential for social desirability bias. Future research could incorporate more objective measures, such as supervisor-evaluated creativity, to improve rigor and credibility.

Variables: First, this study offers a novel approach for examining the conditions under which challenge stress is positively associated with work creativity. The current model considers only a single third-party variable. Future research could employ multiple moderation analyses to simultaneously investigate two or more contextual or individual factors that may shape the relationship between challenge stress and creativity. Furthermore, different forms of creativity may vary in sensitivity to influencing factors. Therefore, creativity may manifest in diverse forms, from incremental innovations to breakthrough ideas, and the relationship between challenge stress and various types of creativity may exhibit more nuanced patterns.

## 9. Conclusions

This study demonstrates that the joint levels of challenge stress and problem-solving pondering are systematically associated with work creativity. Specifically, creativity increased as both challenge stress and problem-solving pondering increased along the CS = PSP reference line. When the relative levels of the two variables differed, a significant asymmetrical U-shaped pattern emerged: creativity was substantially lower when challenge stress exceeded problem-solving pondering, whereas the decline was less pronounced when problem-solving pondering exceeded challenge stress.

Furthermore, cognitive flexibility mediated the relationship between the joint effects of challenge stress and problem-solving pondering and work creativity. By applying the strategy–situation fit perspective and response surface analysis, this study offers a more nuanced explanation of the challenge stress–creativity relationship. Practically, organizations should focus not only on increasing challenging work demands but also on cultivating employees’ problem-solving coping and cognitive flexibility to foster workplace innovation.

## Figures and Tables

**Table 1:** Descriptive statistics and correlation matrix of research variables.

	CS (T1)	PSP (T1)	CF (T2)	C (T2)
CS (T1)	1			
PSP (T1)	0.26 **	1		
CF (T2)	0.13 **	0.19 **	1	
C (T2)	0.01	0.19 **	0.46 **	1
*M*	16.38	15.17	39.94	16.43
*SD*	5.40	3.55	8.08	3.54

Note. *N* = 568. CS = challenge stress, PSP = problem-solving pondering, CF = cognitive flexibility, C = creativity, ** *p* < 0.01. T1 = Time 1, T2 = Time 2.

**Table 2:** Results of confirmatory factor analysis of hypothetical and competing models.

Models	*χ* ^2^	*df*	*χ*^2^/*df*	CFI	TLI	GFI	RMSEA
four-factor model CS, PSP, CF, C	443.57	217	2.04	0.97	0.97	0.94	0.04
three-factor model CS + PSP, CF, C	1043.33	218	4.79	0.91	0.89	0.86	0.08
two-factor model CS + PSP, CF + C	1931.98	220	8.79	0.81	0.78	0.77	0.12
one-way model CS + PSP + CF + C	3496.19	221	15.82	0.64	0.59	0.61	0.16

Note. *N* = 568. CS = challenge stress, PSP = problem-solving pondering, CF = cognitive flexibility, C = creativity.

**Table 3:** Quadratic polynomial regression analysis of the joint effects of challenge stress and problem-solving pondering on work creativity.

	M1		M2		M3	
	*B*	*SE*	*B*	*SE*	*B*	*SE*
age	−0.01	0.02	−0.01	0.02	−0.02	0.02
gender	−0.64 *	0.30	−0.62 *	0.30	−0.57	0.30
tenure	−0.01	0.02	−0.01	0.02	−0.01	0.02
Education 2	1.07 **	0.34	1.00 **	0.34	1.01 **	0.34
Education 3	1.43 *	0.67	1.18	0.66	1.25	0.66
elderly	0.35	0.31	0.47	0.31	0.48	0.31
job type 2	−0.60	0.47	−0.51	0.46	−0.48	0.46
job type 3	−0.69	0.46	−0.82	0.46	−0.74	0.46
job type 4	−1.80	0.89	−1.70	0.88	−1.70	0.87
child care	−0.80 *	0.32	−0.78 *	0.31	−0.90 **	0.31
weekly working hours	−0.01	0.02	−0.02	0.02	−0.01	0.02
CS b_1_			−0.04	0.03	−0.05	0.03
PSP b_2_			0.19 ***	0.04	0.22 ***	0.04
CS^2^ b_3_					0.00	0.00
CS × PSP b_4_					−0.03 **	0.01
PSP^2^ b_5_					0.01	0.01
R^2^	0.06		0.09		0.11	
ΔR^2^	0.06 **		0.03 ***		0.02 **	
Response Surface Parameters						
CS = PSP reference line						
slope (a_1_ = b_1_ + b_2_)					0.16 **	0.05
curvature (a_2_ = b_3_ + b_4_ + b_5_)					−0.02	0.01
CS = −PSP reference line						
slope (a_3_ = b_1_ − b_2_)					−0.27 ***	0.05
curvature (a_4_ = b_3_ − b_4_ + b_5_)					0.03 **	0.01

Note. *N* = 568. Challenge Stress (CS); Problem Solving Pondering (PSP); * = *p* < 0.05; ** = *p* < 0.01; *** = *p* < 0.001. For job type, state-owned enterprises were used as the reference category. For education, junior college degree or below was used as the reference category.

**Table 4:** Results of multiple regression analyses with creativity as the dependent variable.

Variable	CF		Creativity	
	*B*	*SE*	*B*	*SE*
block	1.00 ***	0.19	0.19 *	0.08
CF			0.18 ***	0.02
age			−0.01	0.02
gender			−0.37	0.28
tenure			−0.01	0.02
Education 2			0.62	0.31
Education 3			0.76	0.61
elderly			0.36	0.28
job type 2			−0.41	0.42
job type 3			−0.51	0.42
job type 4			−1.09	0.81
child care			−0.58	0.29
weekly working hours			−0.03	0.02

Note. *N* = 568. Cognitive Flexibility (CF); * = *p* < 0.05; *** = *p* < 0.001. For job type, state-owned enterprises were used as the reference category. For education, junior college degree or below was used as the reference category.

## Data Availability

The data presented in this study are available upon request from the first author. The data are not publicly available because of privacy policy restrictions.
